# Carboxylated or Aminated Polyaniline—Multiwalled Carbon Nanotubes Nanohybrids for Immobilization of Cellobiose Dehydrogenase on Gold Electrodes

**DOI:** 10.3390/bios4040370

**Published:** 2014-10-22

**Authors:** Johannes Tanne, Daniel Kracher, Birgit Dietzel, Burkhard Schulz, Roland Ludwig, Fred Lisdat, Frieder W. Scheller, Frank F. Bier

**Affiliations:** 1 Fraunhofer Institute for Cell Therapy and Immunology (IZI-BB), Branch Bioanalytics and Bioprocesses Potsdam-Golm, Am Mühlenberg 13, 14476 Potsdam, Germany; E-Mail: fschell@uni-potsdam.de; 2 Food Biotechnology Laboratory, Department of Food Sciences and Technology, BOKU-University of Natural Resources and Life Sciences, Muthgasse 18, 1190 Vienna, Austria; E-Mails: daniel.kracher@boku.ac.at (D.K.); roland.ludwig@boku.ac.at (R.L.); 3 Institute for Thin Film and Microsensoric Technology, Kantstr. 55, 14513 Teltow, Germany; E-Mails: b.dietzel@gmx.net (B.D.); buschu@uni-potsdam.de (B.S.); 4 Biosystems Technology, Institute of Applied Life Sciences, Technical University of Applied Sciences Wildau, Hochschulring 1, 15745 Wildau, Germany; E-Mail: flisdat@th-wildau.de

**Keywords:** multiwalled carbon nanotubes, polyaniline, nanohybrids, cellobiose dehydrogenase

## Abstract

Polymer-multiwalled carbon nanotube (MWCNT) nanohybrids, which differ in surface charge have been synthesized to study the bioelectrocatalysis of adsorbed cellobiose dehydrogenase (CDH) from *Phanerochaete sordida* on gold electrodes. To obtain negatively charged nanohybrids, poly(3-amino-4-methoxybenzoic acid-co-aniline) (P(AMB-A)) was covalently linked to the surface of MWCNTs while modification with *p*-phenylenediamine (PDA) converted the COOH-groups to positively charged amino groups. Fourier transform infrared spectroscopy (FTIR) measurements verified the *p-*phenylenediamine (PDA) modification of the polymer-CNT nanohybrids. The positively charged nanohybrid MWCNT-P(AMB-A)-PDA promoted direct electron transfer (DET) of CDH to the electrode and bioelectrocatalysis of lactose was observed. Amperometric measurements gave an electrochemical response with K_Mapp_ = 8.89 mM and a current density of 410 nA/cm^2^ (15 mM lactose). The catalytic response was tested at pH 3.5 and 4.5. Interference by ascorbic acid was not observed. The study proves that DET between the MWCNT-P(AMB-A)-PDA nanohybrids and CDH is efficient and allows the sensorial detection of lactose.

## 1. Introduction

Substrate detection with electrochemical biosensors allows the sensitive and selective measurement of analytes. Biosensors consist of a transducer and a recognition element, which is selective for the analyte. The immobilization method of the redox active enzyme to the electrode strongly influences the analytical performance. An efficient electron transfer is relevant for the communication between the electrode and the analyte-specific enzyme to obtain a defined signal and to minimize interferences from the matrix. Mediators can act as an electron shuttle between the enzyme and the transducer. Biosensors, which use electron mediators, often show high current densities and a good sensitivity. However, direct electron transfer (DET) between electrode and enzyme can compensate several obstacles of mediator-based second generation biosensors, e.g., diffusion of mediators, expensive heavy metal complexes or toxicity of the shuttle molecule. This explains the high interest in the DET of redox enzymes as a basis for third-generation biosensors. For DET a direct connection or an electron pathway between the electrode surface and the prosthetic group of the enzyme has to be established. While the active site of the redox active enzyme has to be orientated towards the electrode, the substrate transport and conversion should not be influenced by the immobilization method. 

Biosensors of the third generation can be used to determine substrates at low concentrations. Heme proteins such as cyt *c*, myoglobin or hemoglobin are used to detect superoxide anion, antioxidants [1,2,3,4], nitrite, trichloroacetic acid [5] or nitric oxide [6,7,8], respectively. Peroxidases like horseradish peroxidase (HRP) can also be used for DET by electroreduction of H_2_O_2_ [9,10]. 

Self-assembled monolayers are often used for modifying the surface and promoting DET when it comes to the immobilization of the recognition element. For example alkyl thiols can chemisorb on the surface of gold electrodes, expose hydrophilic or hydrophobic groups towards the solution to generate an efficient interface for the catalytic protein. Besides covalent coupling or direct adsorption several other immobilization methods have been developed to fix the biocatalyst in a productive way, e.g., affinity binding [11], Langmuir Blodgett films [12], entrapment [13], multilayer assemblies [14] or molecular wires [15]. 

Nanomaterials increase the active surface area drastically. This allows the immobilization of a higher amount of enzymes in comparison to a flat surface [16,17]. Furthermore, many nanomaterials can easily be modified. Gold nanoparticles can be treated with thiols or thiol-modified DNA [18], silver nanoparticles increase the Raman-effect [19], platinum-particles are good catalysts for H_2_O_2_ [20,21], quantum dots allow the light-triggered read-out of a current signal [22,23] and carbon nanotubes (CNTs) have a good conductivity and functional groups for modification or protein binding [24,25]. 

One challenging issue in manufacturing a new generation of biosensors is the immobilization of the redox enzyme to the surface of the CNTs for DET. The enzyme can be adsorbed via electrostatic or hydrophobic interactions; it can be covalently coupled to functional groups, entrapped within a polymer or bond through affinity binding. However, DET becomes inefficient if the distance between the active center and the CNT surface is too long. To improve the contact, chemical-engineering techniques of the enzyme are one possibility, e.g., by deglycolysation [26,27]. However this variation of the protein structure may lead to a decrease of enzyme activity or stability.

Another way is the premodification of the CNTs with conducting polymers. The polymers can facilitate the electron transport through interaction with the enzyme. For example glucose dehydrogenase was immobilized with substituted sulfonated polyanilines on gold electrodes allowing DET [28]. The conducting polymers can also be bound to the surface of CNTs via electropolymerization [29]. Polypyrrol was deposited on single-walled carbon nanotubes (SWCNTs) in the presence of horseradish peroxidase [30] or nitrite reductase [31], and the DET was established between the enzymes and the SWCNTs. 

Alternatively, electropolymers have also been used as a simple immobilization matrix with no role in transferring electrons to the SWCNTs [32,33,34]. In such a structure the polymer improves the immobilization while the CNTs increase the overall conductivity and decrease the operation potential. This allows the measurement of slow oxidation processes and may suppress interferences by easily oxidizable substances, e.g., ascorbic acid or uric acid [30,35]. 

Different kinds of nanohybrids have been synthesized by covalent coupling of sulfonated polyaniline to carboxylic acid groups of the CNTs [36,37]. A terpolymer grafted to multiwalled carbon nanotubes has recently been used in a biosensor. The nanohybrid showed an increase in the protein coverage of cyt *c* in comparison to unmodified polymer-multiwalled carbon nanotubes (MWCNTs). By increasing the degree of sulfonation, the peak current of the redox protein could also be enhanced. The study showed that the immobilization of cyt *c* was possible and that a more efficient DET to the nanostructured electrode could be achieved [38].

The first studies of cellobiose dehydrogenase (CDH) on nanostructured electrodes used single-walled carbon nanotubes (SWCNT) for the adsorption of the enzyme. The SWCNTs were modified by chemical oxidation and adsorbed after drop-casting on a glassy carbon (GC) electrode. After covalent coupling, an increase in the current signal compared to unmodified electrodes could be detected [39]. Other enzymes like glucose oxidase (GOD) have also been immobilized on CNT-modified electrodes [40,41,42].

In this study we demonstrate the use of differently charged polymer-CNT nanohybrids for an enzymatic system. CDH was chosen as model enzyme because of its unique properties. It consists of two domains—an FAD containing domain where the sugar oxidation takes place and a heme *b*-containing domain, which shuttles the electrons from the FAD domain to an external electron acceptor. Therefore, two different ways exist for electron transfer: Whereas small mediator molecules can pick up electrons directly from the FAD domain, interaction with surfaces is dominated by internal electron transfer to the heme domain and from there to the electrode [43]. Thus, DET can be studied by using the redox properties of the cytochrome to charge and discharge the biocomponent and in the catalytic mode by generating a steady current in the presence of an electron donor like lactose. In this study, CDH was immobilized on amino- or carboxy-modified polymer CNT nanohybrids on gold electrodes and the electron transfer was studied.

## 2. Experimental Section

### 2.1. Reagents

Aniline, 3-amino-4-methoxybenzoic acid, ammonium persulfate (APS), HCl, DMF, and sodium hydroxide, oxalyl chloride, *N*-(3-dimethylaminopropyl)-*N*′-ethylcarbodiimide hydrochloride (EDC), *N*-hydroxysuccinimide (NHS), acetic acid, mercaptopropionic acid, cysteamine, *p-*phenylenediamine and lactose were bought from Sigma-Aldrich (Steinheim, Germany). MWCNT-COOH (approx. 9 nm diameter; 1.5 µm length; 4% carboxylated) was purchased from Nanocyl (S.A., Belgium).

### 2.2. Electrode Cleaning

Au electrodes (2 mm Gold Working Electrode, CH Instruments, Llanelli, UK) were polished with Al_2_O_3_ powder with decreasing grain size (0.3, 0.05 µm, Leco, Michigan, USA) for 4 min on a nylon cloth. Afterwards the electrodes were sonicated for 10 min (35 Hz; Bandelin Sonorex, Berlin Germany). In the next step the electrodes were electrochemically cleaned with cyclic voltammetry in 1 M NaOH (−800 mV to +200 mV *vs*. Ag/AgCl, 1 M KCl, 300 mVs^−1^) and in 0.5 M H_2_SO_4_ (−250 mV to +1.75 V *vs*. Ag/AgCl, 1 M KCl, 300 mVs^−1^). The electrodes were rinsed after each step with ultrapure water and additionally by ethanol after the last cycling and stored under dry conditions. 

### 2.3. Electrode Modification

Cleaned electrodes were incubated in 20 mM cysteamine or 20 mM mercaptopropionic acid (MPA) in water overnight at 4 °C. Afterwards the electrodes were washed five times in water. MWCNT-P(AMB-A) or MWCNT-P(AMB-A)-PDA were sonicated for 30 min at 35 kHz (Bandelin Sonorex Super 10P) in 50 mM MES pH 5.5. The sonicated MWCNT-P(AMB-A) or MWCNT-P(AMB-A)-PDA (in 50 mM MES pH 5.5) were used to dilute 50 mM EDC and 100 mM sulfo-NHS. MWCNT-P(AMB-A) or MWCNT-P(AMB-A)-PDA were coupled to the cysteamine or MPA-modified gold electrodes, respectively. The electrodes were incubated for at least 2 h at 300 rpm on a cycler (peqlab Thriller). After several washing steps in 100 mM acetate buffer (pH 3.5) 10 µL of CDH from *Phanerochaete sordida* (9.63 mg/mL; 162 U/mL, produced according to the method from Ludwig *et al.* [44]) were dropped on the nanostructured electrodes. For control experiments CDH was adsorbed on the cysteamine or MPA-modified electrodes. The pH for the measuring buffer was adjusted with NaOH to pH 3.5 or pH 4.5.

### 2.4. Synthesis of Poly(3-Amino-4-Methoxybenzoic Acid-co-Aniline) P(AMB-A)

Three-amino-4-methoxybenzoic acid (3.3 g, 0.02 mol) was dissolved in 100 mL 1.2 M HCl at 50 °C. Aniline (1.86 g, 0.02 mol) and ammonium peroxodisulfate (13.7 g, 0.066 mol) dissolved in 50 mL 1.2 M HCl were added. The solution was stirred for 2 h at 50 °C and 20 h at room temperature. The precipitated product was filtered, washed with aqueous 1.2 M HCl, water and acetone and dried under vacuum at 80 °C for 24 h. 

### 2.5. MWCNTs Functionalized with P(AMB-A)

**Figure 1:**
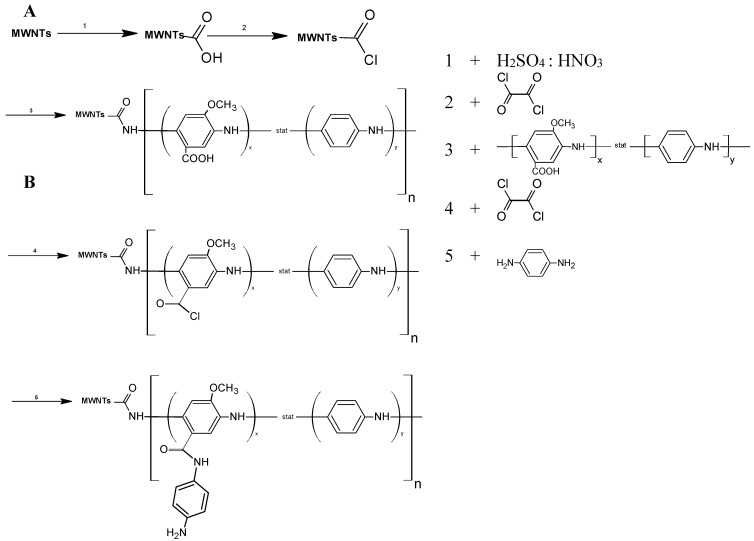
Schematic pathway for covalent coupling of poly(3-amino-4-methoxybenzoic acid-co-aniline) (P(AMB-A)) to polymer-multiwalled carbon nanotubes (MWCNTs) (**A**) and functionalization of P(AMB-A) with *p*-phenylenediamine (PDA) (**B**).

As described in the literature, the carboxylic acid groups of modified MWCNTs were converted into an acyl chloride by treatment with oxalyl chloride [36,37]. The acyl chloride-activated carboxy groups of the MWCNTs reacted by an amidation reaction with poly(3-amino-4-methoxybenzoic acid-co-aniline) (Figure 1A). 

### 2.6. Synthesis of MWNT-P(AMB-A)-PDA

An amount of 0.5 g MWCNT-P(AMB-A) in 500 mL DMF was sonicated to give a homogeneous suspension. Twenty milliliters of oxalyl chloride were added dropwise to the MWCNT-P(AMB-A) suspension at 0 °C for 2 h at room temperature for another 4 h and at 70 °C for 20 h. Finally the temperature was raised to 100 °C for 2 h to remove excess oxalyl chloride. Two grams of *p*-phenylenediamine dissolved in 40 mL DMF were added and stirred for 20 h at 100 °C. After cooling to room temperature, the reaction mixture was separated by centrifugation and washed several times with DMF, ethanol, water, and acetone and dried under vacuum until a constant weight was reached (Figure 1B).

### 2.7. Measurements

#### 2.7.1. Fourier Transform Infrared Spectroscopy (FTIR)

Infrared (IR) spectra were recorded on a Bio-Rad FTX 3000MX spectrometer, in the range of 600–3500 cm^−1^.

#### 2.7.2. Cyclic Voltammetry (CV).

All electrochemical measurements were done in a home-made 2 mL cell (PTFE) using a gold electrode as working electrode (2 mm diameter), an Ag/AgCl, 1 M KCl reference (CH Instruments, UK) and a Pt-wire counter electrode. Cyclic voltammetry experiments were carried out with the CH Instruments Electrochemical workstation (CHI, UK). The potential range was chosen between −0.1 and +0.6 V with a scan rate of 1 mVs^−1^. Data analysis was performed using the original 9.0 peak and baseline analyzer.

#### 2.7.3. Lactose Detection

Au-MPA-[MWCNT-P(AMB-A)-PDA]/CDH or Au-cysteamine-[MWCNT-P(AMB-A)]/CDH were placed into a measuring chamber and acetate buffer was added. The current was measured until a stable signal was reached (*ca*. 100 s) in 100 mM acetate buffer pH 3.5. Lactose was added and the change of the current was investigated until a steady state current (I_ssc_) was reached. The solution was permanently stirred. For measurements at higher pH value the acetate buffer was set to pH 4.5 with NaOH. The studies were done at either +100 mV, 0 mV or −100 mV (*vs.* Ag/AgCl, 1 M KCl). For the determination of potential interference, ascorbic acid was added into the measuring cell in a range between 50 µM and 1 mM in 100 mM acetate buffer (pH 4.5) at 0 mV (*vs*. Ag/AgCl, 1 M KCl). Before the addition of ascorbic acid, the response of the biosensor for 1 mM lactose was measured to ensure that the sensor was working.

## 3. Results and Discussion

### 3.1. Synthesis of the P(AMB-A) and Coupling to the Surface of MWCNTs

**Figure 2:**
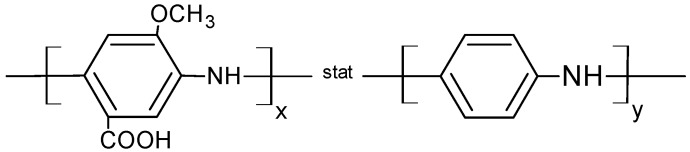
Chemical structure of poly(3-amino-4-methoxybenzoic acid-co-aniline) P(AMB-A).

To study the performance of CDH from *Phanerochaete sordida*, two different nanohybrids were synthesized. These nanohybrids consisted of a polyelectrolyte covalently bound to the surface of multiwalled carbon nanotubes. Modified polyaniline was chosen because of its good conducting properties [45]. The copolymer was synthesized by the polymerization of anilin (A) and 3-amino-4-methoxybenzoic acid (AMB). The polyelectrolyte has a molecular weight of about 4.7 kDa. The methoxylation leads to an electron donating effect within the polymer and therefore increases its electron density [46]. Methoxylated polyanilines have already been used in biosensors and could improve electron transfer (ET) [47]. The carboxylation makes the polymer soluble and it can also be used to couple the polymer to the surface of amino-functionalities. Figure 2 shows the statistical formula of P(AMB-A).

The synthesized polymer was covalently coupled to the carboxylic acid groups of multiwalled carbon nanotubes. For this purpose the MWCNTs were activated with oxalyl chloride. In order to change the properties of the polymer-CNT-nanohybrid a second system was prepared by modifying the carboxylic groups in the polymer with *p-*phenylenediamine (PDA) to provide amino groups.

**Figure 3:**
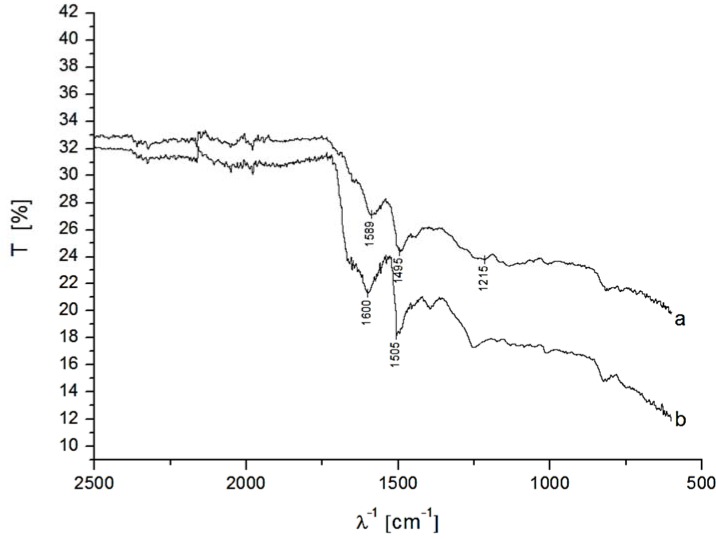
Fourier transform infrared spectroscopy (FTIR)-spectra of MWCNT-P(AMB-A) (**a**) and MWNT-P(AMB-A)-PDA (**b**).

The two different nanohybrids were analyzed by fourier transform infrared spectroscopy (FTIR) measurements. Figure 3 shows the FTIR-spectra of the modified MWCNT-P(AMB-A) and MWCNT-P(AMB-A)-PDA. The vibration modes of the benzene rings (quinoid and benzoid) appear at 1495 and 1589 cm^−1^ for MWCNT-P(AMB-A) and are slightly shifted to 1505 and 1600 cm^−1^ for MWCNT-P(AMB-A)-PDA. The bands at 1215 and 1688 cm^−1^ in the IR-spectrum for MWCNT-P(AMB-A) caused by the aromatic COOH group disappear in the spectrum of MWCNT-P(AMB-A)-PDA showing the successful amidation reaction [48]. At 1640 cm^−1^ a shoulder is visible in both spectra caused by the vibrations of an amide bond of N–H [36,49] which is increased after the PDA modification. 

### 3.2. Analysis of DET from *Phanerochaete sordida* CDH on Different Nanohybrid-Modified Gold Electrodes

In the following electrochemical studies it was analyzed whether CDH could be immobilized to the different nanohybrids and whether DET from CDH to the electrode was feasible. First the carboxy-modified nanohybrids were used for the enzyme immobilization. 

In order to couple MWCNT-P(AMB-A) to the electrode a self-assembled monolayer (SAM) of cysteamine was prepared on gold and the MWCNT-P(AMB-A) nanohybrid was covalently bound with EDC/NHS activation. In a next step, the enzyme was adsorbed to the electrode and the response was measured in acetate buffer at pH 3.5. In presence of 5 mM lactose no catalytic current could be detected. It is known that the IET of CDH in solution is strongly dependent on the pH and decreases dramatically above 4.5. At pH 5 only 34% of the enzymatic activity remain and at pH 5.5 only 8% remain [50]. An additional experiment was also performed at pH 4.5, but no response was detected. Either the protein cannot bind to the MWCNT-P(AMB-A)-modified electrode or the orientation of the enzyme is not suitable for the electron transfer. 

**Figure 4:**
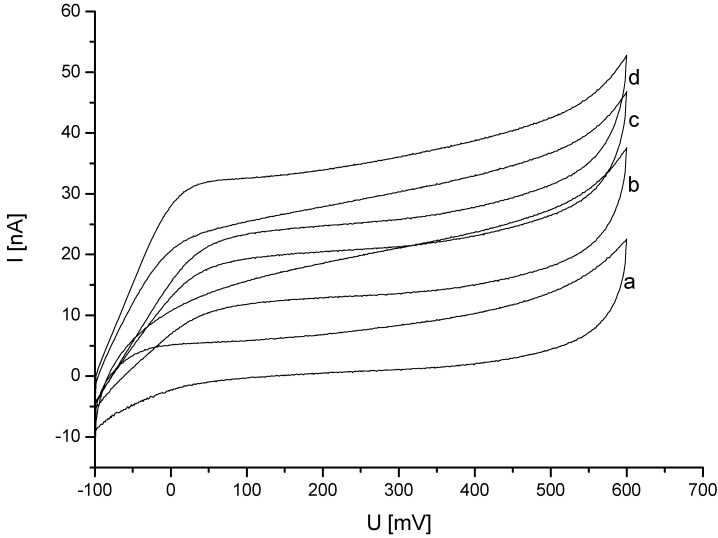
Au-MPA-[MWCNT-P(AMB-A)-PDA]/CDH in (**a**) 100 mM acetate buffer pH 3.5 and addition of (**b**) 5 mM (**c**) 10 mM (**d**) 15 mM lactose (1 mVs^−1^; *vs*. Ag/Ag/Cl, 1 M KCl).

In an alternative approach, the amino-containing nanohybrids based on the PDA modification were tested as an interface for the enzyme. For this purpose mercaptopropionic acid (MPA) was bound to purified gold electrodes. The thiol layer provides carboxylic groups, which can be activated with EDC/NHS. Afterwards MWCNT-P(AMB-A)-PDA was covalently coupled via the amino-groups of the PDA-modification. Subsequently, CDH was adsorbed onto the nanostructured surface. The electrodes were measured in acetate buffer to investigate the charging current in the cyclic voltammogram (CV). No redox peaks of the enzyme were visible at that low scan rate. After addition of lactose, the measurement was repeated. In Figure 4 the CV measurements for different lactose concentrations are shown. A clear increase in the oxidative current was visible after the substrate was added. 

In comparison to carboxylated nanohybrids the PDA-modified nanohybrids allow electron transfer from the enzyme to the electrode. This is mainly attributed to the different charges on the nanohybrids. 

The isoelectric point (pI) of the heme domain is not known. However, the overall pI of CDH from *Phanerochaete sordida* is 4.1 and the pI of the FAD domain is 5.7 [50]. For CDH from *Phanerochaete chrysosporium* the values have been determined to be 5.45 for the FAD part and 3.42 for the heme part [51]. Therefore, a pI below 3.5 of the heme domain of CDH from *Phanerochaete sordida* can be assumed. The measurements of Au-MPA-[MWCNT-P(AMB-A)-PDA]/CDH were carried out at pH 3.5. Also in previous studies a pH of 3.5 was chosen for the electrochemical measurement of CDH from *Phanerochaete sordida* [50,52]. At this pH, the FAD domain is positively charged and the heme domain should be slightly negatively charged. We suppose that the immobilization of CDH on MWCNT-P(AMB-A)-PDA takes place via the heme domain, which allows the electrical communication with the positively charged nanohybrid. The fact that the potential at which DET starts is in the range of the redox potential of the heme iron center supports this assumption. *Vice versa*, the positively charged FAD domain might be oriented towards negatively charged nanohybrids through electrostatic interaction. This situation would not result in an efficient direct electron transfer.

Studies of CDH adsorbed at different surfaces show that the correct orientation of the heme domain to the nanostructured surface is important for the electron transfer. This most probably explains why there is no catalytic current at MWCNT-P(AMB-A) nanohybrids. 

Most of the biosensors use the DET communication between the heme domain and the electrode so that the IET from the FAD to the heme becomes an important factor. Previous studies of CDH from *Phanerochaete sordida* on SWCNT and COOH-modified SWCNT resulted in a catalytical current after lactose was added. However the current response was the same for both systems [50,53]. Therefore the negative charges may not be beneficial for enhancing bioelectrocatalysis. The oxidation potential was also around the redox potential of the heme iron. After modification of SWCNTs with amino groups and subsequent adsorption of CDH, Tasca *et al*. measured a 3.5-fold increase in the catalytical current. The authors suggested that the high current signal resulted from the good DET communication between the heme domain and the derivatisation of the SWCNTs. Their electrocatalytic response has been the highest signal for a lactose biosensor so far. In absence of the substrate, Tasca *et al.* could measure a redox wave at −50 mV (*vs*. NHE). But the peaks were caused by FAD released from the protein and gave no additional response after addition of lactose [50,52,53]. Therefore, up to this point in time, mediator-based systems are the only known contact to the FAD domain of CDH. For example, biosensors based on osmium-polymers—with an oxidation potential below that of the heme domain—on SWCNT modified electrodes allow the transfer of electrons between the FAD domain and an electrode [52]. 

It has been reported that CDH from *Phanerochaete chrysosporium* can be productively immobilized on different SAMs of NH_2_–; COOH– or OH– terminated thiols. Matsumura *et al.* detected a catalytic current at hydrophilic and hydrophobic promoter layers at +100 mV (*vs*. NHE). The thiols had an alkyl chain of 11 C-Atoms. However, they had to cover the electrode with a permselective membrane to retain the CDH at the SAM-modified electrode [54]. In the same way several electrodes are based on CDH at a SAM entrapped by a permselective membrane [55,56,57]. 

**Figure 5:**
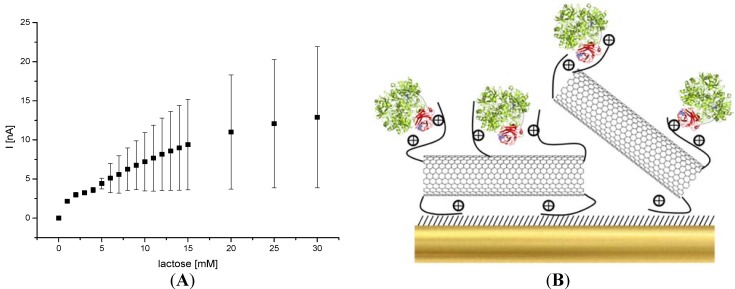
(**A**) Concentration dependence of the amperometric response of an Au-MPA-[MWCNT-P(AMB-A)-PDA]/CDH electrode for lactose (determined from I_ssc_ of measurements at in 100 mM acetate buffer pH 3.5 at +100 mV; *vs*. Ag/AgCl, 1 M KCl). (**B**) Scheme of the CDH assembly on [MWCNT-P(AMB-A)-PDA] modified gold electrode. Heme domain is colored in red while the FAD domain is green.

Since the enzyme can be effectively connected via amino group containing nanotubes the system was applied for amperometric measurements with different lactose concentrations. In Figure 5 the concentration dependence at a potential of +100 mV *vs.* Ag/AgCl is plotted. The figure shows the average of steady state current signals of four electrodes. A typical saturation curve was found. With higher concentrations, a large variation among the electrodes becomes visible. An apparent K_Mapp_ of 8.89 mM was calculated for the immobilized enzyme.

**Figure 6:**
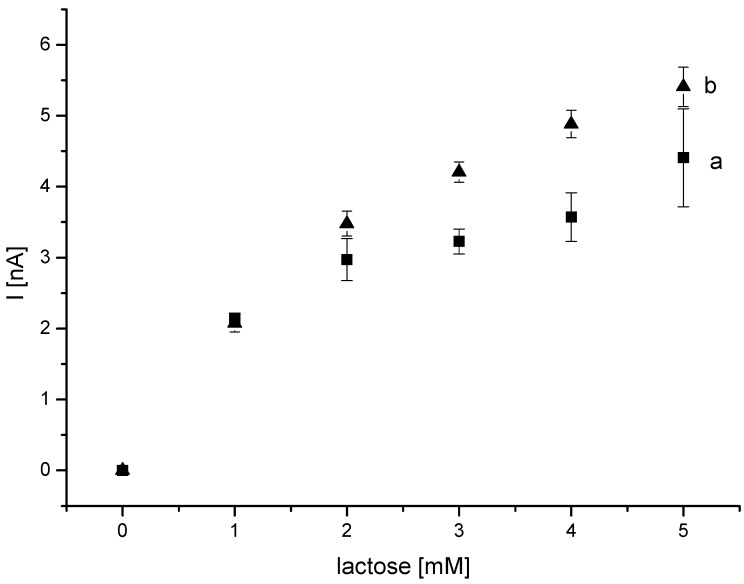
Amperometric current response of Au-MPA-[MWCNT-P(AMB-A)-PDA]/CDH electrode in dependence of the lactose concentration measured at two different pH values: (**a**) pH 3.5 and (**b**) pH 4.5 in acetate buffer at +100 mV (*vs*. Ag/Ag/Cl, 1 M KCl).

The current density of the biosensor at 15 mM lactose is around 410 nA/cm^2^_._ The current is five times higher than that of CDH-biosensors based on screen-printed electrodes with adsorbed polyaniline [58]. The response time of our sensor after lactose addition was rather fast (2 s). 

The DET is only effective up to a pH of 4.5, which corresponds to the IET of *P. sordida* CDH. Thus the pH variation was limited to this value. The current signals of Au-MPA-[MWCNT-P(AMB-A)-PDA]/CDH were compared for pH 4.5 and pH 3.5 (Figure 6). Eight electrodes were prepared in the same way as mentioned in the experimental section. Four electrodes were measured at pH 3.5. The other four electrodes were measured at pH 4.5. At pH 4.5, the enzyme is still active and can convert lactose. No decrease in the current response was observed indicating that both the internal reaction between the domains and the interaction between the heme domain and the electrode are not strongly influenced. 

### 3.3. Study of Potential Ascorbic Acid Interference

**Figure 7:**
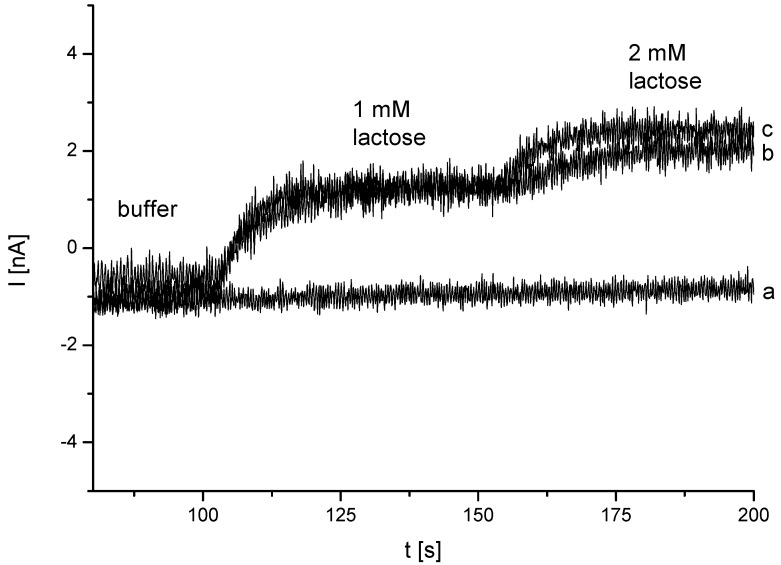
Amperometric measurement of Au-MPA-[MWCNT-P(AMB-A)-PDA]/CDH at (**a**) −100 mV, (**b**) ±0 mV and (**c**) +100 mV (100 mM acetate buffer pH 4.5; *vs*. Ag/Ag/Cl, 1 M KCl).

Many biosensors based on CDH detect lactose at +300 mV (*vs.* Ag/AgCl) or +100 mV (*vs*. Ag) [39,57]. At positive potentials interfering substances can be oxidized at the electrode and may thus interfere with the bioelectrocatalytic signal generation. Previous studies of interference on rotating disk electrodes showed anodic waves at +300 mV (*vs.* Ag/AgCl) for ascorbate, while Trolox, an analog of vitamine E, can be already oxidized at +80 mV (*vs*. Ag/AgCl). Particularly ascorbate and ureate can reach rather high concentrations in dairy products [59]. The biosensor was consequently further analyzed at different potentials in acetate buffer at pH 4.5. Figure 7 shows an amperometric measurement at different potentials after addition of lactose.

At −100 mV (*vs*. Ag/AgCl) no catalytic current of the enzyme electrode could be detected. Measurements at 0 mV (*vs.* Ag/AgCl) showed a clear signal increase after addition of lactose. The current signal was in the same range as for +100 mV (*vs.* Ag/AgCl) (Table 1). This allowed the detection at very low potentials where interfering substances, e.g., uric acid or ascorbic acid do not influence the signal of the lactose measurement. 

**Table 1:** Amperometrical response after addition of 1 mM lactose at different potentials applied to Au-MPA-[MWCNT-P(AMB-A)-PDA]/CDH.

U [mV]	I [nA]
−100	0.04 ± 0.02
±0	1.92 ± 0.085
+100	2.09 ± 0.086

It has to be mentioned that the polymer also may have an effect on the oxidation of ascorbate. Bartlett *et al.* described a sensor for ascorbate based on polyaniline-polyvinylsulfonate composite coated on glassy carbon electrodes. They detected a signal even below 0 mV (*vs*. SCE) [45]. Self-doped polyaniline synthesized from aniline and *o*-aminobenzoic acid could also be used to detect ascorbic acid at ~ +90 mV (*vs.* SCE) [60] or ~ +70 mV (*vs.* SCE) [61]. The concentration of detectable ascorbic acid was in a linear range from 12 µM up to 2.4 mM. 

In order to analyze the behavior of ascorbic acid and to test whether it can interfere with the biosensor response, different concentrations of the molecule were investigated. We choose a range between 50 µM and 1 mM of ascorbic acid in 100 mM acetate buffer and measured at 0 mV (*vs.* Ag/AgCl) in acetate buffer pH 4.5 (data not shown). No influence on the current signal could be detected after the addition of ascorbic acid. One explanation why there is no catalytic response after addition of the interfering substance could be the low concentration of polymer at the polymer-CNT-nanohybrid modified electrode in comparison to a fully covered polyaniline electrode as described in literature and the modification of the polyaniline performed here.

Due to the low oxidation potential, the well-defined response, and negligible interference by ascorbic acid, the biosensor can be used for lactose determination in real samples, e.g., in the dairy industry.

### 3.4. Storage Stability

To test the storage stability of the nanohybrid based enzyme electrodes they were kept over two weeks at 4 °C in acetate buffer when not in use. After addition of 1 mM lactose 85% of the maximum signal were still detected at the end of this period. This means that most of the enzymes are still active on the surface of MWCNT-P(AMB-A)-PDA-modified electrodes and capable of electron transfer to the nanohybrids. It has to be emphasized here that these results have been obtained with the enzyme only adsorbed to the nanostructured surface.

## 4. Conclusions

Nanohybrids of modified polyaniline and MWCNTs seem to be a valuable tool for the immobilisation of redox enzymes in biosensors. The adsorption of CDH from *Phanerochaete sordida* to the positively charged nanohybrids of [MWCNT-P(AMB-A)-PDA] resulted in an efficient bioelectrocatalytic system. With its high sensitivity in acidic pH (corresponding to the optimal IET of CDH), the low operational potential and good storage stability, the biosensor is suitable for lactose determination in dairy products. The nanohybrids may be useful in the future for biosensors with redox enzymes of a similar or less complex surface charge structure.
